# Insulin-Like Growth Factor Axis Biomarkers and Gestational Diabetes Mellitus: A Systematic Review and Meta-Analysis

**DOI:** 10.3389/fendo.2019.00444

**Published:** 2019-07-12

**Authors:** Xi-Rui Wang, Wen-Juan Wang, Xiaodan Yu, Xiaolin Hua, Fengxiu Ouyang, Zhong-Cheng Luo

**Affiliations:** ^1^Ministry of Education–Shanghai Key Laboratory of Children's Environmental Health, Xinhua Hospital, Shanghai Jiao-Tong University School of Medicine, Shanghai, China; ^2^Department of Developmental Pediatrics, Shanghai Children's Medical Center, Shanghai Jiao-Tong University School of Medicine, Shanghai, China; ^3^Department of Obstetrics and Gynecology, Prosserman Centre for Population Health Research, Lunenfeld-Tanenbaum Research Institute, Mount Sinai Hospital, and Institute of Health Policy, Management and Evaluation, Dalla Lana School of Public Health, University of Toronto, Toronto, ON, Canada; ^4^Department of Obstetrics and Gynecology, Xinhua Hospital, Shanghai Jiao-Tong University School of Medicine, Shanghai, China

**Keywords:** insulin-like growth factor, insulin-like growth factor binding protein, gestational diabetes mellitus, systematic review, meta-analysis

## Abstract

The insulin-like growth factor (IGF) axis has been implicated in glucose homeostasis. It is plausible to hypothesize that the IGF axis is involved in the development of gestational diabetes mellitus (GDM). In a systematic review of the evidence on IGF axis biomarkers in relation to GDM, we searched the PubMed and EMBASE for publications up to May 31, 2018, on the associations of circulating IGF axis biomarkers with GDM. Eligible studies must meet the pre-specified quality assessment criteria. Meta-analyses were conducted where there were at least three studies on the same biomarker at the same gestational age window—early (<20 weeks), mid (20–29 weeks), or late (30+ weeks) gestation. Twelve studies were included (484 GDM, 1755 euglycemic pregnancies). Meta-analyses showed that GDM was consistently associated with higher IGF-I concentrations in mid-gestation (six studies) and late gestation (six studies). There were only two studies on IGF-I in early gestation and GDM with inconsistent findings. GDM was associated with lower IGFBP-2 concentrations in early, mid-, or late gestation, according to data from one or two studies. GDM was associated with higher IGFBP-3 concentrations in late gestation according to a meta-analysis of five studies. There was no association with GDM for IGFBP-3 in early or mid-gestation, according to data from one study. Other IGF axis biomarkers (IGF-II, IGFBP-1,−4,−5−6, and −7) showed no or inconsistent associations, and the data at early gestation were scanty or absent. Available evidence is suggestive but inconclusive concerning whether the IGF axis is involved in the development of GDM. More studies on IGF axis biomarkers in early gestation are warranted. If a specific IGF axis molecule is proven to be involved in the development of GDM, this may point to a new molecular target for designing interventions to reduce the incidence of GDM.

## Introduction

Gestational diabetes mellitus (GDM), commonly defined as impaired glucose tolerance with onset or first recognition during pregnancy, affects 5–15% of pregnant women (1, 2). The etiology of GDM remains incompletely understood, but pancreatic β-cell function insufficiency in compensating for pregnancy-induced insulin resistance is thought to be important, resulting in hyperglycemia in the second half of pregnancy (3). GDM develops when the maternal insulin supply is insufficient to maintain euglycemia during pregnancy. GDM increases the risk of maternal complications (gestational hypertension and preeclampsia) and fetal and neonatal complications (congenital malformations, macrosomia, preterm birth, and shoulder dystocia) (4). GDM may also “program” long-term adverse consequences such as the metabolic syndrome, type 2 diabetes, and cardiovascular disease in the offspring (5).

Traditionally linked to the regulation of cellular growth and differentiation, the insulin-like growth factor (IGF) axis is a signal transduction complex consisting of (1) the growth factors (IGF-I and IGF-II), (2) IGF binding proteins (IGFBPs) that may regulate their bio-available fractions, and (3) membrane receptors through which they act (6). Given the structural similarities of IGFs with insulin, IGFs and other components of the IGF axis have been implicated in glucose homeostasis (7, 8). Therefore, it is plausible to hypothesize that the IGF axis is involved in the development of GDM.

The metabolic effects of IGF-I are to provide a signal to cells that adequate nutrients are available to avoid apoptosis and enhance cellular protein synthesis enabling cells to undergo hypertrophy in response to an appropriate stimulus and stimulating cell division. Studies have shown that IGF-I can promote glucose uptake in peripheral tissues (9, 10) and suppress hepatic glucose production (11, 12). A significant positive correlation between insulin sensitivity and endogenous IGF-I concentration in patients with glucose intolerance has been reported (13). We are unaware of any data on whether IGF-II is related to insulin sensitivity.

The IGFBPs may also play a role in glucose metabolism. IGFBP-1 may regulate glucose levels through its impact on free IGF-I level (14). IGFBP-2 has been associated with an anti-diabetic effect in mice (15). IGFBP-3 is the most abundant IGFBP in circulation, and its metabolic effects are largely opposite to IGF-I; IGFBP-3 inhibits the biological activity of IGF-I by sequestrating IGF-I into a circulating reservoir, thereby reducing free IGF-I levels in circulation, and has been positively associated with the risk of diabetes (16).

Given the suggested roles of the IGF axis in glucose homeostasis, it is plausible that maternal circulating concentrations of IGF axis biomarkers may be associated with GDM. To our knowledge, there is no systematic review on the relationships between maternal IGF axis biomarkers and GDM. We thus conducted a systematic review and meta-analysis of the literature on circulating IGF axis biomarkers in relation to GDM, following the MOOSE Guidelines for Meta-Analyses and Systematic Reviews of Observational Studies (17).

## Methods

This was a systematic review and meta-analysis. Before the review, we conducted an initial literature screen in PubMed to affirm that the topic of interest has not yet been systematically reviewed. The review protocol was not registered in any registry.

### Data Sources

We searched the PubMed and EMBASE for publications up to May 31, 2018 (date last searched) on IGF axis biomarkers in relation to GDM using the following keywords: (gestational diabetes mellitus or gestational diabetes or GDM) and (insulin-like growth factor or IGF-I or IGF-II or IGF-1 or IGF-2 or IGF1 or IGF2 or insulin-like growth factor binding protein or IGF binding protein or IGFBP). There was no language restriction on publications. We did not use the keyword IGF receptor since exploratory searches did not find any report on circulating IGF receptors in GDM (probably undetectable in circulation). A total of 282 article titles were retrieved. Two reviewers (XRW, a PhD candidate in pediatrics; and WJW, a PhD candidate in perinatal epidemiology) independently screened all titles and abstracts for relevance (i.e., whether the study addresses the associations of maternal circulating IGF axis biomarkers with GDM). Discrepancies were resolved through discussions with a senior reviewer (XY, professor in pediatrics; XH, associate professor in obstetrics; FO, professor in pediatric epidemiology; Z-CL, scientist in perinatal epidemiology). Review articles were considered relevant in this initial literature screening. A total of 72 abstracts were deemed relevant, and the full-length articles were obtained for further assessment of eligibility. Bibliographies of retrieved articles were cross-referenced to identify additional studies. This review did not cover unpublished studies, which might be of uncertain quality.

### Study Selection

Eligible studies must meet all of the following criteria: (1) studies must contain original data on maternal IGF axis biomarkers in relation to GDM in humans, (2) observational studies (cross-sectional, case–control, or cohort studies), and (3) plasma or serum concentrations of IGF axis biomarkers available. We excluded review articles (9), studies measuring IGF axis biomarkers from inappropriate blood samples (following stimulation or collected in the non-pregnancy period, *n* = 21), studies that did not separate GDM from chronic diabetes (*n* = 13), and studies with cases only (*n* = 5), leaving 24 articles for study quality assessment.

The primary studies were assessed using pre-defined quality assessment criteria for non-randomized observational studies adapted from Duckitt and Harrington (18) with some modifications to match the needs of the present systematic review. The assessment items included the representativeness of study participants, comparability of groups, definition of outcome, ascertainment of outcome, sample size, and study design (Table 1). Two reviewers independently conducted the quality assessment, and any differences were resolved through discussions with a senior third reviewer (Z-CL, XH, or FO).

**Table 1:** Quality assessment of non-randomized observational studies.

**1. Selection of participants (1/0)**
*Cohort studies* (1/0) Selected cohort was representative of the general population (population-based studies) or target catchment (hospital-based studies) population (1) Cohort was a selected unrepresentative group or the selection of the group was not defined (0) *Case–control studies* (1/0) Cases and controls drawn from the same population (1) Cases and controls drawn from different sources or the selection of groups was not described (0)
**2. Comparability of groups (2/0)** No significant differences between the groups reported in terms of age and pre-existing medical conditions were explicitly reported, or these differences were adjusted for in the analyses (2). Differences between groups were not examined (1). Groups differed and no adjustment results provided (0)
**3. Definition of outcomes (2/0)** Definition of outcomes (gestational diabetes) Referenced definition or explicit specified commonly accepted definition (2) Explicit modified definition, but according to commonly accepted definition (1) Unspecified or unacceptable definition (0)
**4. Ascertainment of outcomes (2/0)** How the diagnosis was made Prospectively diagnosed or review of notes/hospital discharge records (2) ICD or database coding (1) Process not described (0)
**5. Sample size (1/0)** ≥300 participants in a cohort study, or ≥20 in each study group in a case control study (1) <300 participants in a cohort study, or <20 in either study group in a case control study (0)
**6. Study design (2/0)** Prospective (2) Cross-sectional or retrospective (1) Not described or poorly designed (0)
**Exclusion:** score zero in any item (1 to 6) or a total score <7 out of 10 maximal points

**Figure 1:**
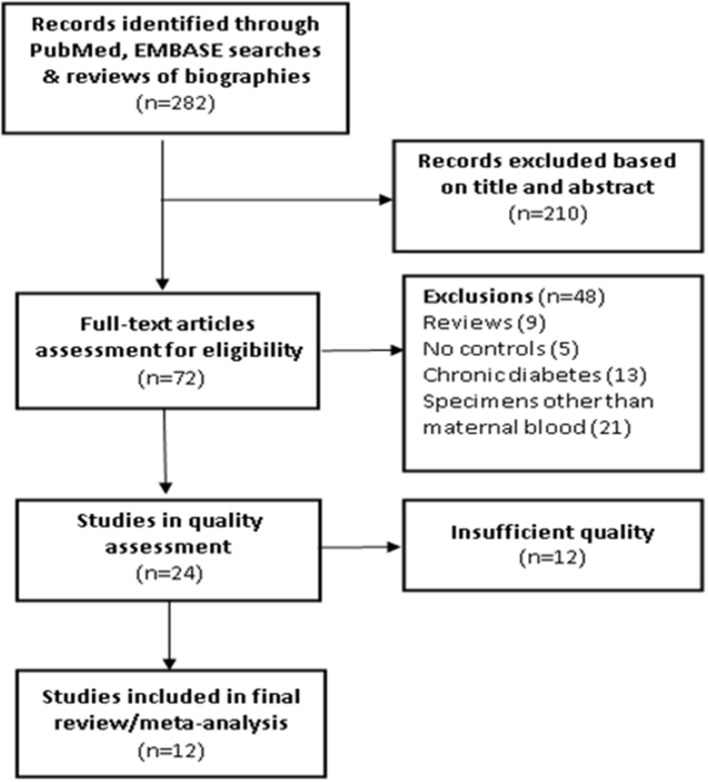
Flowchart in the selection of studies in a systematic review of maternal circulating IGF axis biomarkers and gestational diabetes mellitus (GDM).

We excluded studies that scored zero in any of the six quality assessment categories or with a total score <7 out of 10 maximal points. A total of 12 original studies were retained in the final systematic review (19–30); their quality scores are presented in Table 2. Among these studies, 7 studies scored 10, 3 studies scored 9, and 2 studies scored 8. The flowchart in the selection of studies is presented in Figure 1.

**Table 2:** Quality assessment scores of 12 studies included in the systematic review of circulating IGF axis biomarkers and gestational diabetes mellitus (GDM).

**References**	**Selection participants**	**Comparability of groups**	**Outcome**		**Sample size**	**Study design**	**Score**
			**Definition**	**Ascertainment**			
Luo et al. (22)	1	2	2	2	1	2	10
Qian et al. (23)	1	1	2	2	1	2	9
Hayati et al. (24)	1	1	2	2	1	2	9
Matuszek et al. (19)	1	2	2	2	1	2	10
O'Leary and Longley (26)	1	2	1	1	1	2	8
Ramirez et al. (20)	1	2	2	2	1	2	10
Zhu et al. (27)	1	2	2	2	1	2	10
Grissa et al. (21)	1	2	2	2	1	2	10
Lappas (25)	1	2	2	2	1	1	9
Hughes et al. (29)	1	2	1	2	1	1	8
Qiu et al. (28)	1	2	2	2	1	1	10
Liao et al. (30)	1	1	2	2	1	1	10

### Data Extraction, Tabulation, and Analysis

Two reviewers independently extracted relevant study data from the original articles, and discrepancies were resolved through discussions with a senior reviewer (XY, XH, FO, or Z-CL). The following information was extracted into an Excel spreadsheet: the first author, country, year of publication, maternal race/ethnicity, age, body mass index (BMI), definition of GDM, method in the ascertainment of GDM, study design, sample size, comparability of groups, gestational age at blood sampling, type of maternal blood specimen (plasma or serum), and mean and standard deviation (SD) of the reported plasma or serum concentrations of IGF axis biomarkers (IGF-I, IGF-II, and IGFBPs). Where the SD was unavailable but the standard error (SE) available, the SD was calculated from the SE (SD = SE multiplied by the square root of sample size). Where the required data are unclear or unavailable, we contacted the corresponding author through email for clarification in at least two attempts 2 weeks apart.

Data were summarized for IGF axis biomarkers in early (≤ 19 weeks, before the GDM diagnosis), middle (20–29 weeks, around the time of GDM diagnosis; GDM is routinely screened at 24–28 weeks of gestation, although some high-risk patients may be screened earlier), and late pregnancy (30+ weeks, after the diagnosis and/or treatment) separately. Summary statistics were calculated using Review Manager 5.3 (Cochrane Collaboration, Oxford, United Kingdom). The inverse variance method was adopted in the meta-analysis to calculate the weighted mean difference (WMD) and 95% confidence intervals (CI) comparing GDM vs. euglycemic (control) women. The heterogeneity between studies was indicated intuitively by the *I*^2^ statistics. If *I*^2^ > 50%, a random effects model was used in the pooled data analysis; otherwise, a fixed effects model was applied. When there were less than three study data lines in a comparison, we did not conduct pooled data meta-analysis, but described the key results in individual studies.

Where the odds ratios (ORs) or relative risks (RRs) were reported, we described them in individual studies. Because the ORs or RRs were often unavailable, and were calculated according to different approaches (e.g., the RRs comparing the highest vs. lowest quartiles, or the highest vs. lowest tertiles), we did not calculate the pooled OR or RR.

### Patient and Public Involvement

There is no patient and public involvement in this systematic review.

## Results

### Characteristics of Included Studies

Of the 12 studies included in the systematic review (total: 484 GDM and 1,755 euglycemic/control pregnancies), 4 studies were from North America, 2 from Australia, and 6 from other countries (Table 3). Caucasian was the most commonly studied race. There were four prospective cohort studies, three nested case–control studies, and five case control studies. There were eight studies measuring IGF axis biomarkers in maternal serum and four studies in maternal plasma.

**Table 3:** Characteristics of included studies in a systematic review of insulin-like growth factor (IGF) axis biomarkers and GDM.

**References**	**Country**	Study type^*^	**GDM/Control**	**Ethnicity**	**Population**	**Age (years)**	**BMI (kg/m^**2**^)**	**Definition of GDM**	**GA (weeks) at blood sampling**	**Specimen**
Ramirez et al. (20)	USA	PC	30/42	Hispanic (92%)	Obese	GDM 30.9 ± 4.7 NGT 28.1 ± 5.4	GDM 34.1 ± 4.5 NGT 34.4 ± 4.7	ADA 100 g OGTT	24–28	Serum
Matuszek et al. (19)	Poland	CC	46/21	Caucasian	General	GDM 29 (28-32) NGT 29 (26-33)	GDM 23.2 NGT 21.6	PDA 75 g OGTT	24–28	Serum
Zhu et al. (27)	USA	NCC	107/214	Multi-ethnic	General	GDM 30.5 ± 5.7 NGT 30.4 ± 5.4	GDM 28.2 ± 6.4 NGT25.6 ± 5.3	ACOG 100 g OGTT	10–14, 15–26, 32–39	Plasma
Lappas (25)	Australia	CC	44/30	Caucasian	Non-obese	GDM 34.8 ± 5.3 NGT 33.3 ± 4.4	GDM 25.0 ± 3.3 NGT 24.3 ± 3.8	ADPS	At delivery	Plasma
	Australia	CC	26/36	Caucasian	Obese	GDM 33.8 ± 4.6 NGT 32.3 ± 4.2	GDM 36.9 ± 6.6 NGT 37.8 ± 6.0	75 g OGTT		
Luo et al. (22)	Canada	PC	27/280	Caucasian	General	GDM 31 ± 4.7 NGT 30.8 ± 4.7	GDM 25.3 ± 5.9 NGT 23.4 ± 4.6	ADA 75 g OGTT	24–28; 32–35	Plasma
Grissa et al. (21)	Tunisia	CC	30/30	Tunisian	General	19–42	GDM 24.9 ± 2.9 NGT 23.2 ± 2.3	FBG≥5.5	At delivery	Serum
Hayati et al. (24)	Malaysia	CC	25/50	Asian	General	NA	NA	WHO 75 g OGTT	28, 36	Serum
O'Leary and Longley (26)	Australia	NCC	34/200	Caucasian	General	NA	NA	ADA 75 g OGTT	28–35	Serum
Qian et al. (23)	China	CC	20/38	Asian	General	22–34	NA	75 g OGTT	At delivery	Serum
Qiu et al. (28)	USA	PC	47/757	White	General	≥35: 27.9%	≥30: 8.7%	ADA 100 g OGTT	13	Plasma
Hughes et al. (29)	Britain	PC	20/29	NA	General	GDM 30 (19-42) NGT 26 (20-38)	NA	WHO 75 g OGTT	31–40	Serum
Liao et al. (30)	New Zealand	NCC	28/28	Caucasian	General	GDM 31.4 ± 4.8 NGT 31.3 ± 4.2	GDM 27.2 ± 4.8 NGT 26.2 ± 4.2	IADPSG 75 g OGTT	20	Serum

* *Study type: PC, prospective cohort study; NCC, nested case–control study; CC, case–control study. GA, gestational age; GDM, gestational diabetes mellitus; NGT, normal glucose tolerance; FBG, fasting blood glucose; ADA, American Diabetes Association; ACOG, American College of Obstetricians and Gynecologists; ADPS, Australia Diabetes in Pregnancy Society; IADPSG, International Association of Diabetes and Pregnancy Study Groups; PDA, Polish Diabetes Association; WHO, World Health Organization; NA, not available*.

### IGF Axis Biomarkers

IGF-I was reported in all the 12 studies, while IGF-II was reported in only 3 studies (Table 4). IGFBP-1,−2, and −3 were reported in five, two, and six studies, respectively (Table 5), while there was only one study on IGFBP-4,−5,−6, and −7 (25). We did not conduct country- or race-specific subgroup analyses due to the small number of studies.

**Table 4:** Summary data in studies on circulating IGF-I and IGF-II levels^a^ in GDM and control (euglycemic) pregnancies.

Study type^*^	**References**	**Country**	**Specimen**	**Assay**	**GA**.	**GDM (ng/ml)**^**a**^			**Controls (ng/ml)**^**a**^			**GDM vs. control difference (95% CI)^**c**^**
				**Method**	**Weeks**	***N***	**Mean**	***SD***	***N***	**Mean**	***SD***	
**IGF-I**												
PC	Luo et al. (22)	Canada	Plasma	ELCA	24–28	27	285.6	109.5	280	200.4	80.4	**85.20 (42.84, 127.56)**
PC	Luo et al. (22)	Canada	Plasma	ELCA	32–35	27	403.6	171.8	279	307.0	123.6	**96.60 (30.19, 163.01)**
PC	Hayati et al. (24)	Malaysia	Serum	ELISA	28	25	300	90	50	254	127	**46.00 (2.85, 89.15)**
PC	Hayati et al. (24)	Malaysia	Serum	ELISA	36	25	389	85	50	302	106	**87.00 (42.58, 131.42)**
PC	Matuszek et al. (19)	Poland	Serum	ELISA	24–28	46	152.6	87.5	21	120.8	58.8	31.75 (−3.91, 67.41)
PC	Hughes et al. (29)	Britain	Serum	RIA	31–40	20	416	92	29	296	83	**120.00 (69.62, 170.38)**
PC	Ramirez et al. (20)	USA	Serum	ELISA	26	30	173.96	65.04	42	197.0	191.1	−23.04 (−85.34, 39.26)
NCC	Zhu et al. (27)	USA	Plasma	ELISA	10–14	107	180.2	64.1	214	164.9	57.4	**15.27 (0.90, 29.64)**
NCC	Zhu et al. (27)	USA	Plasma	ELISA	15–26	107	217.5	92.2	214	181.9	70.3	**35.61 (15.76, 55.46)**
NCC	Zhu et al. (27)	USA	Plasma	ELISA	32–35	38	335.8	579.1	45	293.1	144.8	42.78 (−146.13, 231.69)
NCC	Zhu et al. (27)	USA	Plasma	ELISA	37–39	51	326.5	302.0	58	297.1	216.3	29.41 (−70.43, 129.25)
PC	Grissa et al. (21)	Tunisia	Serum	ELISA	Delivery	30	650.9	168.0	30	414.3	100.6	**236.66 (166.59, 306.73)**
CC	Lappas (25)	Australia	Plasma^&^	ELISA	Delivery	44	58.0	35.8	30	57.4	25.7	0.60 (−13.42, 14.62)
CC	Lappas (25)	Australia	Plasma^&^	ELISA	Delivery	26	55.1	40.8	36	51.8	31.2	3.30 (−15.40, 22.00)
CC	Qian et al. (23)	China	Serum	RIA	Delivery	20	239.85	68.9	38	201.5	52.4	38.35 (3.86,72.84)
NCC	Liao et al. (30)	New Zealand	Serum	ELISA	20	28	275.7	60.9	28	218.5	58.7	50.20 (25.87, 88.53)
PC	Qiu et al. (28)	USA	Plasma	ELISA	13	47	NA	NA	757	NA	NA	Categorical data only
**Meta-Analysis**^**b**^												
GA	<20 weeks	2 studies										<3 studies
	20–29	6 studies	*P* <0.001									**42.12 (28.87, 55.37)**
	30+	6 studies	*P* <0.001									**98.07 (47.23, 148.90)**
**IGF-II**												
PC	Luo et al. (22)	Canada	Plasma	ELCA	24–28	27	864.6	164.3	280	905.0	143.2	−40.40 (−104.6, 23.80)
PC	Luo et al. (22)	Canada	Plasma	ELCA	32–35	27	983.5	244.8	279	995.7	180.4	−12.20 (−106.93, 82.53)
CC	Lappas (25)	Australia	Plasma^&^	ELISA	Delivery	44	293.7	189.0	30	269.5	175.8	24.20 (−67.43, 115.83)
CC	Lappas (25)	Australia	Plasma^&^	ELISA	Delivery	26	309.9	245.8	36	267.8	210.0	42.10 (−86.66, 170 86)
NCC	Liao et al. (30)	New Zealand	Serum	ELISA	20	28	814.7	131.8	28	836.4	100.5	−21.70 (−83.09, 39.69)
**Meta-Analysis**^**b**^												<3 studies

*Some studies reported maternal IGF-I and/or IGF-II data at multiple gestational age windows, and thus occupied multiple data lines in the table*.

* *Study type: PC, Prospective Cohort study; NCC, Nested Case Control study; CC, Case-Control study; NA, not available. GA, gestational age (weeks); ELISA, enzyme-linked immunosorbent assay; ELCA, enzyme-labeled chemiluminescent assay; RIA, radioimmunoassay*.

a *Conversion factor: IGF-I, 1 nmol/L = 7.649 μg/L or ng/ml; IGF-II, 1 nmol/L = 7.469 μg/L or ng/ml*.

b *There was only one study on free IGF-I or free IGF-II (Lappas 2015 study); all other studies are on total IGF-I or total IGF-II; meta-analysis was conducted on total IGF-I or IGF-II in circumstances with ≥3 studies*.

c *The differences with 95% CIs excluding the zero are shown in bold*.

& *The study on free IGF-I or free IGF-II*.

**Table 5:** Summary data in studies on circulating IGFBP-1, IGFBP-2, and IGFBP-3 levels in GDM and control pregnancies.

Study type^*^	**References**	**Pub year**	**Country**	**Specimen**	**Assay**	**GA**	**GDM (ng/ml)**			**Controls (ng/ml)**			**GDM vs. control difference (95% CI)^**b**^**
					**Method**	**Weeks**	***N***	**Mean**	***SD***	***N***	**Mean**	***SD***	
**IGFBP-1**													
NCC	O'Leary and Longley (26)	1994	Australia	Serum	RIA	28–35	34	146	105	200	135	199	11.00 (−25.88, 47.88)
PC	Ramirez et al. (20)	2014	USA	Serum	ELISA	26	30	44.9	18.8	42	62.2	21.2	–**17.28 (**–**26.57**, –**7.99)**
NCC	Liao et al. (30)	2017	New Zealand	Serum	ELISA	20	28	41.04	18.1	28	67.58	32.6	−26.54 (−40.35, 12.73)
CC	Lappas (25)	2015	Australia	Plasma	ELISA	Delivery	44	54.4	33.8	30	72.3	41.1	−17.90 (−35.77,−0.03)
CC	Lappas (25)	2015	Australia	Plasma	ELISA	Delivery	26	56.2	39.3	36	41.1	31.8	15.10 (−3.23, 33.43)
PC	Qiu et al. (28)	2005	USA	Plasma	ELISA	13	47	NA	NA	757	NA	NA	Categorical data only
**Meta-Analysis**^**a**^													<3 studies
													In all the 3 GA periods
**IGFBP-2**													
CC	Lappas (25)	2015	Australia	Plasma	ELISA	Delivery	44	157.8	109.4	30	189.3	126.5	−31.50 (−87.12, 24.12)
CC	Lappas (25)	2015	Australia	Plasma	ELISA	Delivery	26	150.3	131.6	36	146.4	125.4	3.90 (−61.19, 68.99)
NCC	Zhu et al. (27)	2016	USA	Plasma	ELISA	10–14	107	91.61	50.44	214	116.99	47.67	–**25.38 (**–**36.87**, –**13.89)**
NCC	Zhu et al. (27)	2016	USA	Plasma	ELISA	15–26	107	82.26	27.90	214	103.82	41.16	–**21.56 (**–**29.20**, –**13.92)**
NCC	Zhu et al. (27)	2016	USA	Plasma	ELISA	32–35	38	78.57	29.36	45	88.92	29.36	−10.35 (−23.03, 2.33)
NCC	Zhu et al. (27)	2016	USA	Plasma	ELISA	36–39	51	87.44	15.53	58	91.87	15.53	−4.43 (−10.27, 1.41)
**Meta-Analysis**^**a**^													<3 studies
													In all the 3 GA periods
**IGFBP-3**													
NCC	Zhu et al. (27)	2016	USA	Plasma	ELISA	10–14	107	4638.4	892.7	214	4513.9	858.2	124.5 (−80.0, 329.0)
NCC	Zhu et al. (27)	2016	USA	Plasma	ELISA	15–26	107	4648.6	819.2	214	4561.2	872.1	87.4 (−106.9, 281.7)
NCC	Zhu et al. (27)	2016	USA	Plasma	ELISA	32–35	38	5034.3	1596.0	45	5120.0	2089.9	−85.7 (−879.6, 708.2)
NCC	Zhu et al. (27)	2016	USA	Plasma	ELISA	36–39	51	5085.7	1602.9	58	4965.7	1922.5	120.0 (−542.1, 782.1)
PC	Grissa et al. (21)	2010	Tunisia	Serum	ELISA	Delivery	30	1836.5	912.9	30	1302.6	912.8	**533.9 (71.9, 995.9)**
PC	Hughes et al. (29)	1995	Britain	Serum	RIA	31–40	20	6612.5	1918.7	29	5546.8	1403.5	**1065.7 (81.8, 2049.6)**
CC	Lappas (25)	2015	Australia	Plasma	ELISA	Delivery	44	3997.4	996.3	30	3693.7	1081.2	303.7 (−182.5, 789.9)
CC	Lappas (25)	2015	Australia	Plasma	ELISA	Delivery	26	3959.9	1433.3	36	4029.0	1487.4	−69.1 (−803.7, 665.5)
CC	Qian et al. (23)	2000	China	Serum	RIA	Delivery	20	5676	1628	38	5746	1512	−70.0 (−930.3, 790.3)
NCC	Liao (30)	2017	New Zealand	Serum	ELISA	20	28	5189.2	1783.8	28	5297.3	1270.3	−108.1 (−919.2, 703.0)
**Meta-Analysis**^**a**^													
GA	<20 weeks	1	Study										<3 studies
	20–29	2	Study										<3 studies
	30+	5	Studies	*P* = 0.02									**282.15 (39.78, 524.53)**

* *Study type: PC, prospective cohort study; NCC, nested case–control study; CC, case–control study; NA, not available. GA, gestational age; ELISA, enzyme-linked immunosorbent assay; ELCA, enzyme-labeled chemiluminescent assay; RIA, radioimmunoassay*.

a *Meta-analysis was conducted in circumstances with ≥3 studies*.

b *The differences with 95% CIs excluding the zero are shown in bold*.

Publication bias was assessed by Funnel plot when there were at least three studies on the same biomarker. There was no evidence of publication biases in all reported IGF axis biomarkers (data not shown).

### Early Gestation (<20 Weeks)

There were only two studies on IGF axis biomarkers in early gestation in relation to subsequent development of GDM. Zhu and colleagues reported a nested case–control study on total IGF-I, IGFBP-2, IGFBP-3, and IGF-I/IGFBP-3 ratio in 107 GDM and 214 euglycemic control women (27). IGF-I concentration at 10–14 weeks of gestation was positively associated with subsequent development of GDM. Compared to the highest vs. lowest quartiles in IGF-I concentration, there was a 2.87-fold increased risk of GDM after adjusting for major risk factors (*RR* = 2.87, 95% CI 1.28–6.42, *P* = 0.02). A similar association was observed for the molar ratio of IGF-I to IGFBP-3. However, IGFBP-3 itself was not associated with GDM. A strong negative association was observed between IGFBP-2 and GDM; the highest quartile at 10–14 weeks was associated with a 95% reduced risk of GDM (RR = 0.05, 95% CI 0.02–0.16, *P* < 0.001).

Qiu et al. studied 804 women in a prospective cohort to analyze plasma concentrations of free IGF-I and IGFBP-1 at 13 weeks of gestation in relation to subsequent development of GDM (28). They found that both free IGF-I and IGFBP-1 were inversely associated with GDM. Women with free IGF-I in the highest tertile (≥1.08 ng/ml) experienced a 69% reduced risk of GDM (*RR* = 0.31, 95% CI 0.12–0.75, *P* = 0.01) compared to women with concentrations in the lowest tertile (<0.80 ng/ml). There was a 57% decreased risk of GDM among women with IGFBP-1 in the highest tertile (≥68.64 ng/ml) (*RR* = 0.43, 95% CI 0.18–1.05), but the association was marginal (*P* = 0.059).

### Mid-gestation (20–29 Weeks)

#### IGF-I

**Figure 2:**
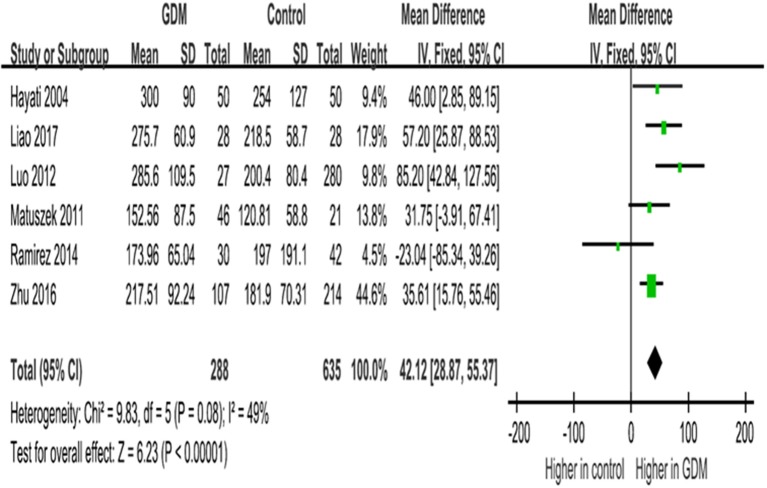
Mean differences (95% CIs) of maternal circulating *total IGF-I concentrations (ng/ml) in mid-gestation (20–29 weeks)* comparing GDM vs. euglycemic (control) pregnancies in six studies. Positive values denote higher values in GDM patients; negative values denote higher values in control subjects.

IGF-I concentrations in mid-gestation were elevated in GDM vs. euglycemic pregnancies, with na WMD of 42.1 ng/ml (95% CI 28.9–55.4, *P* < 0.0001), according to data from six studies (Table 4; Figure 2).

#### IGF-II

There were two studies on IGF-II in mid-gestation and GDM. Luo et al. reported similar plasma total IGF-II concentrations at 24–28 weeks of gestation in GDM and euglycemic women (22). Similarly, Liao and colleagues reported no difference in serum IGF-II concentrations between GDM and control women (30).

#### IGFBPs

There were three studies on IGFBP-1, IGFBP-2, or IGFBP-3 concentrations in mid-pregnancy comparing GDM vs. euglycemic women. Ramirez and colleagues reported significantly lower IGFBP-1 concentrations in GDM vs. controls (mean: 44.9 ± 18.8 vs. 62.2 ± 21.2 ng/ml, *P* = 0.0004) (20). Zhu et al. reported a significant reduction in IGFBP-2 concentrations at 15–26 weeks of gestation in GDM vs. controls (mean: 82.3 ± 27.9 vs. 103.8 ± 41.2 ng/ml, *P* < 0.0001), but similar IGFBP-3 concentrations (mean: 4648.6 ± 819.2 vs. 4561.2 ± 872.1 ng/ml, *P* = 0.38) (27). Liao et al. reported a significant reduction in IGFBP-1 concentrations at 20 weeks of gestation in GDM vs. controls (mean: 41.0 ± 18.1 vs. 67.6 ± 32.6 ng/ml, *P* < 0.001) (30), but similar IGFBP-3 concentrations (mean: 5189.2 ± 1783.8 vs. 5297.3 ± 1270.3 ng/ml, *P* = 0.35) (30).

### Late Gestation (30+ Weeks)

#### IGF-I

**Figure 3:**
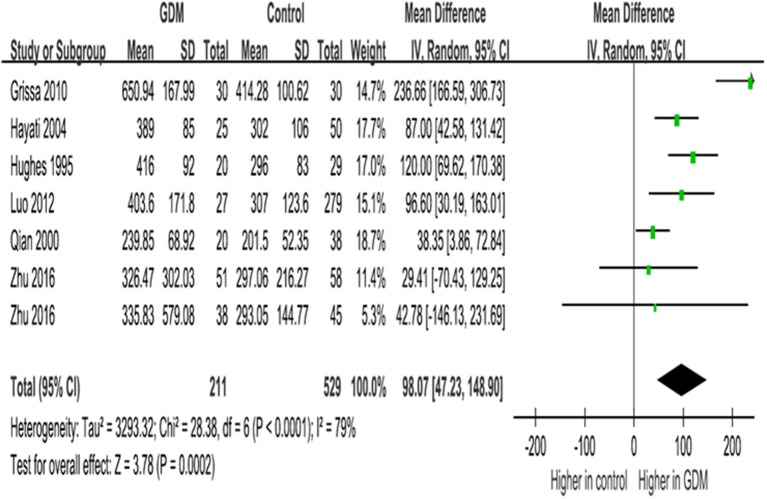
Mean differences (95% CIs) of maternal circulating *total IGF-I concentrations (ng/ml) in late gestation (30*+ *weeks)* comparing GDM vs. euglycemic pregnancies in six studies (seven data lines). Positive values denote higher values in GDM patients; negative values denote higher values in control subjects.

In late gestation, women with GDM had significantly higher IGF-I concentrations (WMD = 98.1 ng/ml, 95% CI 47.2–148.9, *P* = 0.0002), according to data on circulating IGF-I concentrations at ≥30 weeks of gestation in six studies (seven data lines, Figure 3).

#### IGF-II

There were two studies on IGF-II concentrations in late pregnancy and GDM; both did not find an association between IGF-II and GDM (22, 25).

#### IGFBP-1

There were two studies on maternal IGFBP-1 concentrations in late gestation (25, 26); both reported no significant differences in GDM vs. euglycemic pregnancies.

#### IGFBP-2

There were significantly lower IGFBP-2 concentrations in late gestation compared GDM to euglycemic pregnancies (WMD = −5.64 ng/ml, 95% CI −10.90 to −0.37, *P* = 0.04), according to data from two studies (four data lines, Table 5).

#### IGFBP-3

**Figure 4:**
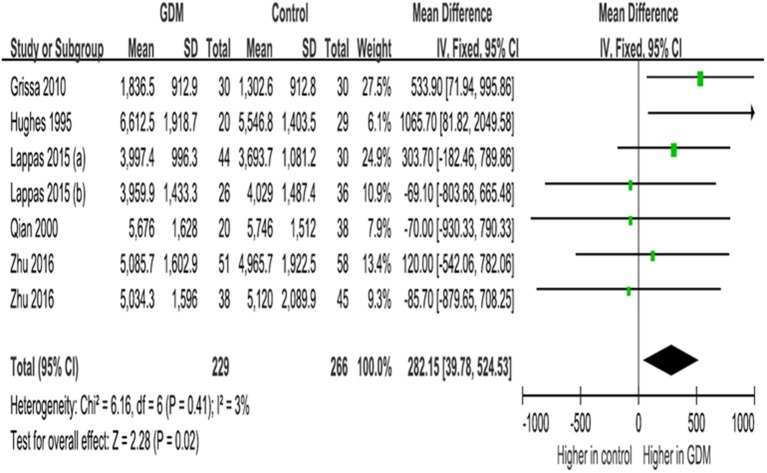
Mean differences (95% CIs) of maternal circulating *IGFBP-3 concentrations (ng/ml) in late gestation (30*+ *weeks)* comparing GDM vs. euglycemic (control) women in five studies (seven data lines). Positive values denote higher values in GDM patients; negative values denote higher values in control subjects.

IGFBP-3 concentrations in late gestation were significantly higher in GDM vs. euglycemic pregnancies (WMD = 282.2 ng/ml, 95% CI 39.8–524.5, *P* = 0.02), according to data from five studies (seven data lines, Figure 4).

#### Other IGFBPs

We are aware of only one study on the associations of GDM with IGFBP-4,−5,−6, or−7, and no association was detected (25).

## Discussion

### Main Findings

To our knowledge, this is the first systematic review on IGF axis biomarkers and GDM. Circulating levels of IGF axis biomarkers are gestational age dependent (22, 27). Thus, gestational age window-specific analyses are critical. This review shows that current research evidence is suggestive but insufficient concerning whether the IGF axis is involved in the development of GDM. GDM was consistently associated with higher IGF-I levels in mid- and late gestation, but there were only two studies in early gestation with inconsistent findings. IGFBP-2 was consistently negatively associated with GDM throughout gestation, but the findings were based on only one or two studies in early, mid-, or late gestation. IGFBP-3 in late gestation was positively associated with GDM, but there was no association for IGFBP-3 in early or mid-gestation according to data from a single study. Other IGF axis biomarkers have shown no or inconsistent associations with GDM, and the data in early gestation are absence or scanty.

The IGF axis has been implicated in glucose homeostasis (7, 8). It is a plausible hypothesis that the IGF axis is involved in the etiology of GDM. If the hypothesis is true, there should be significant alterations in the expression/circulating levels of IGF axis biomarkers before the clinical onset of the disease in early gestation that may or may not persist in mid- and late gestation. Changes in a biomarker can be either a cause or consequence of GDM. GDM is routinely diagnosed around 24–28 weeks of gestation. Only those changes before 20 weeks of gestation could be more confidently considered possibly causal in the development of GDM. Changes in circulating levels of IGF axis biomarkers in mid- and late gestation could be either a cause or consequence of GDM. The scarcity of studies on IGF axis biomarkers at <20 weeks of gestation forestalls a conclusive statement on the etiological role of IGF axis in the development of GDM.

### IGF-I and IGF-II

Under normal physiological conditions, IGF-I has a hypoglycemic effect inhibiting insulin secretion and increasing insulin sensitivity (31). Glucose modulates the secretion of IGF-I through the release of insulin, while IGF-I may regulate insulin levels by a negative feedback (31, 32). Our review suggests that IGF-I levels may be elevated at the time around or after the diagnosis of GDM, but whether it may play a causal role remains uncertain since there were only two studies on IGF-I in early gestation with inconsistent findings (27, 28). The causes of the conflicting findings are unclear, and the solution to resolve the question may be through new and large (adequately powered) prospective pregnancy cohort studies with high-quality biomarker data. The higher IGF-I levels in GDM in mid- and late gestation could be attributable to elevated insulin secretion (33, 34), and/or enhanced secretion of placental growth hormone—the main driver of maternal IGF-I production in pregnancy (35). In contrast, IGF-II appears not to be related to GDM, although caution is warranted in data interpretation since there was only one study on IGF-II in mid-gestation and no study in early gestation.

### IGFBPs

IGFBPs play an important role in insulin signaling, enhancing peripheral glucose uptake and decreasing hepatic glucose output (36). It remains unclear what the differences in the biological significance of various IGFBPs. This review showed that IGFBP-2 was consistently negatively associated with GDM throughout the pregnancy, but the finding was based on one or two studies in early, mid-, or late gestation, and requires confirmation in more independent studies. The negative association between IGFBP-2 and GDM is consistent with the negative association between IGFBP-2 and type 2 diabetes in adults (37). IGFBP-2 over-expression has been associated with reduced susceptibility to obesity and diabetes *via* inhibition of adipogenesis and stimulation of insulin sensitivity in mice (15, 38). The pleiotropic actions of IGFBP-2 suggest its potential as a critical molecule involved in the development of GDM. There is a lack of significant post-prandial fluctuations in IGFBP-2 concentrations (39), rendering IGFBP-2 as a promising early gestational biomarker in predicting the development of GDM, but confirmative studies are wanted.

Our review demonstrates that IGFBP-3 concentrations are elevated in late gestation in women with GDM. In contrast, there are no significant changes in IGFBP-3 concentrations in early or mid-gestation in women who later developed GDM, suggesting that the elevated IGFBP-3 levels may be a consequence, rather than the cause of GDM, but it should be cautioned that there was only one study on IGFBP-3 in early gestation.

There was only one study on IGFBP-1 in early gestation and there were only two studies in mid-gestation; all reported lower IGFBP-1 levels in GDM (20, 28, 30). This is consistent with the finding in adults that higher IGFBP-1 levels are correlated with better glucose tolerance and lower insulin resistance (40). This review also showed that the difference in IGFBP-1 levels disappeared in late gestation. The scanty data in early gestation and inconsistent data in late gestation suggest the need for more studies to clarify the association between IGFBP-1 and GDM.

We identified only one study on IGFBP-4, −5, −6, or −7 concentrations in late gestation in relation to GDM, and the study did not find any significant association (25). There was no study on these IGFBPs in early or mid-gestation.

### Strengths and Weaknesses

The main strength is the coverage of all studies with high-quality original data on circulating IGF biomarkers and GDM; all included studies are of high quality (Table 2). The main weakness is the inability to review the data in the gray literature or unpublished studies, which may be of uncertain quality.

## Conclusions

Current research evidence is suggestive, but limited and insufficient concerning whether the IGF axis is involved in the development of GDM. More studies on IGF axis biomarkers in early gestation and subsequent development of GDM are warranted.

## Author Contributions

Z-CL, XH, XY, and FO conceived the study. X-RW and W-JW conducted the literature search and data extraction. X-RW, W-JW, XH, XY, FO, and Z-CL contributed to the reviews of retrieved papers. X-RW and W-JW conducted the data meta-analysis and drafted the manuscript. All authors contributed to data interpretation, revising the article critically for important intellectual content, and approved the final version for publication.

### Conflict of Interest Statement

The authors declare that the research was conducted in the absence of any commercial or financial relationships that could be construed as a potential conflict of interest.

## Abbreviations

IGF: insulin-like growth factor
IGFBP: IGF binding protein.
